# One-year change in plasma volume and mortality in the Japanese general population: An observational cohort study

**DOI:** 10.1371/journal.pone.0254665

**Published:** 2021-07-13

**Authors:** Yoichiro Otaki, Tetsu Watanabe, Tsuneo Konta, Masafumi Watanabe, Koichi Asahi, Kunihiro Yamagata, Shouichi Fujimoto, Kazuhiko Tsuruya, Ichiei Narita, Masato Kasahara, Yugo Shibagaki, Kunitoshi Iseki, Toshiki Moriyama, Masahide Kondo, Tsuyoshi Watanabe

**Affiliations:** 1 Department of Cardiology, Pulmonology, and Nephrology, Yamagata University School of Medicine, Yamagata, Japan; 2 Steering Committee of Research on Design of the Comprehensive Health Care System for Chronic Kidney Disease (CKD) Based on the Individual Risk Assessment by Specific Health Check, Fukushima, Japan; Universidad Miguel Hernandez de Elche, SPAIN

## Abstract

**Background:**

Changes in plasma volume, a marker of plasma volume expansion and contraction, are gaining attention in the field of cardiovascular disease because of its role in the prevention and management of heart failure. However, it remains unknown whether a 1-year change in plasma volume is a risk factor for all-cause, cardiovascular, and non-cardiovascular mortality in the general population.

**Methods and results:**

We used a nationwide database of 134,291 subjects (age 40–75 years) who participated in the annual “Specific Health Check and Guidance in Japan” check-up for 2 consecutive years between 2008 and 2011. A 1-year change in plasm volume was calculated using the Strauss–Davis-Rosenbaum formula. There were 220 cardiovascular deaths, 1,001 non-cardiovascular deaths including 718 cancer deaths, and 1,221 all-cause deaths during the follow-up period of 3.9 years. All subjects were divided into quintiles based on the 1-year change in plasma volume. Kaplan–Meier analysis demonstrated that the highest 5^th^ quintile had the greatest risk among the five groups. Multivariate Cox proportional hazard regression analysis demonstrated that a 1-year change in plasma volume was an independent risk factor for all-cause, cardiovascular, non-cardiovascular, and cancer deaths. The addition of a 1-year change in plasma volume to cardiovascular risk factors significantly improved the C-statistic, net reclassification, and integrated discrimination indexes.

**Conclusions:**

Here, we have demonstrated for the first time that a 1-year change in plasma volume could be an additional risk factor for all-cause, cardiovascular, and non-cardiovascular (mainly cancer) mortality in the general population.

## Introduction

Heart failure has a high mortality rate and remains a major and increasing public health problem [1]. It has been reported that imbalanced volume homeostasis causes systemic congestion and peripheral and pulmonary edema in patients with heart failure [2]. Plasma volume expansion underlies systemic congestion, which is a well-known, clinically, and prognostically relevant complication of heart failure [3]. The European Society of Cardiology guidelines recommend that blood volume status be assessed in heart failure patients [4]. Heart failure stages range from stage A heart failure (subject at high risk for heart failure) to stage D heart failure (advanced heart failure). Since stage A includes hypertension, diabetes mellitus, and atherosclerotic disease without structural heart disease, health checkups play a pivotal role in the early identification of individuals at high risk for heart failure.

The clinical application of plasma volume assessment has been limited because accurate measurement of plasma volume is technically difficult and invasive because its determination requires pulmonary artery catheterization or administration of tracer molecules [5–7]. Thus, several formulae have been derived from routinely collected clinical data to calculate plasma volume estimates. Although plasma volume calculated using these formulae is a useful predictor of clinical outcome in patients with heart failure [8, 9], these estimates of plasma volume have been reported to correlate poorly with measured plasma volume [10]. Thus, attention has shifted from actual plasma volume estimation to changes in plasma volume.

Changes in plasma volume are calculated using the Strauss–Davis–Rosenbaum formula, and have been reported to be associated with cardiovascular disease in patients with heart failure [9]. Recently, it has been suggested that a change in plasma volume could be a surrogate marker for actual plasma volume change during sodium-glucose co-transporter-2 treatment in patients with diabetes mellitus [11]. However, the impact of a 1-year change in plasma volume on all-cause, cardiovascular, and non-cardiovascular deaths in the general population remains unknown. Thus, we hypothesized that a 1-year change in plasma volume serves as an early identification of high-risk subjects for all-cause, cardiovascular, and non-cardiovascular deaths in the general population. The present study aimed to examine whether a 1-year change in plasma volume is a novel risk factor for all-cause, cardiovascular, and non-cardiovascular deaths in the general population.

## Method

### Ethics statement

All procedures performed in studies involving human participants were undertaken in accordance with the ethical, institutional, and/or national research committee guidelines of the centers at which the studies were conducted (Yamagata University, 2008, no. 103) and in compliance with the 1964 Helsinki declaration and its later amendments or comparable ethical standards. The institutional ethics committee of Yamagata University School of Medicine approved the study.

This study was performed according to the Ethical Guidelines for Medical and Health Research Involving Human Subjects enacted by the Ministry of Health, Labour and Welfare of Japan (http://www.mhlw.go.jp/file/06-Seisakujouhou-10600000-Daijinkanboukouseikagakuka/0000069410.pdf; http://www.mhlw.go.jp/file/06-Seisakujouhou-10600000-Daijinkanboukouseikagakuka/0000080278.pdf). In the context of the guideline, the investigators shall not necessarily be required to obtain informed consent, but we publicized information concerning this study on the web (http://www.fmu.ac.jp/univ/sangaku/data/koukai_2/2771.pdf) and ensured that there was an opportunity for the research subjects to decline the use of their personal information. All data were fully anonymized.

### Study population

**Fig 1:**
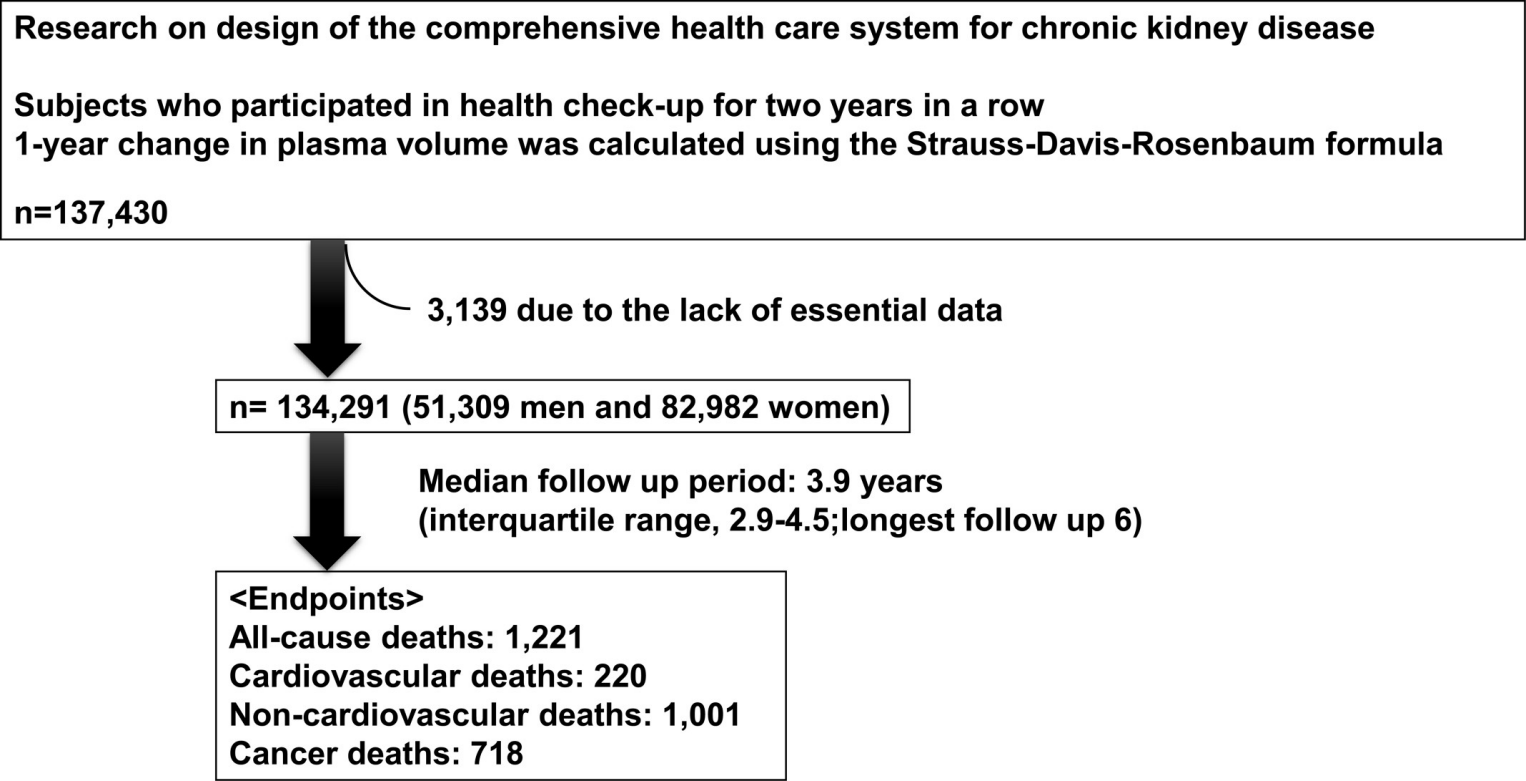
Flow chart of the study selection process.

This study is part of an ongoing “Research on design of the comprehensive health care system for chronic kidney disease (CKD)” based on individual risk assessments by the Specific Health Check-up for all inhabitants of Japan between the ages of 40 and 74 years, and is covered by the Japanese National Health Insurance. We utilized data obtained from the following 20 prefectures (i.e., administrative regions): Hokkaido, Fukushima, Ibaraki, Tochigi, Saitama, Chiba, Nagano, Niigata, Ishikawa, Fukui, Gifu, Osaka, Hyogo, Tokushima, Fukuoka, Saga, Nagasaki, Kumamoto, Miyazaki, and Okinawa. These prefectures were divided into four regions as follows: Hokkaido and Tohoku, Kanto and Koshinetsu, Kinki, Shikoku, and Chugoku, and Kyushu and Okinawa. A flow chart of the selection process used in this study is shown in Fig 1. We collected data from 137,430 subjects (aged 40–74 years) who participated in the health check-ups for 2 consecutive years between 2008 and 2011. Among them, 3,139 were excluded from this study due to lack of essential data. Therefore, 51,309 men and 82,982 women were finally included in this study. Previous cardiovascular disease, previous cerebrovascular disease, previous anemia, and new onsets of cardiovascular disease and cerebrovascular disease were determined using a self-questionnaire.

### Cardiovascular risks and Framingham risk score

Blood pressure was measured using the following method [12]. Participants were seated with back support. After resting for at least 5 min, blood pressure was measured twice without conversation. Blood pressure was determined by an average of two blood pressure readings. Hypertension was defined as a systolic blood pressure ≥ 140 mmHg, diastolic blood pressure ≥ 90 mmHg, or antihypertensive medication use. Diabetes mellitus was defined as a fasting blood sugar (FBS) level of ≥ 126 mg/dL, glycosylated hemoglobin A1c (HbA1c) ≥ 6.5% (National Glycohemoglobin Standardization Program), or anti-diabetic medication use. Dyslipidemia was defined as high-density lipoprotein cholesterol (HDL-C) < 40 mg/dL, low-density lipoprotein cholesterol (LDL-C) ≥ 140 mg/dL, triglyceride ≥ 150 mg/dL, or lipid-lowering medication use. The Framingham risk score was calculated according to a previous report [13].

### Definition of 1-year change in plasma volume and plasma volume status

A 1-year change in plasma volume was estimated according to the Strauss–Davis–Rosenbaum formula: 1-year change in plasma volume = ((baseline hemoglobin/hemoglobin at 1 year)×((1-hematocrit at 1 year)/(1-baseline hematocrit))-1) ×100 [8, 14]. An abnormally large 1-year expansion in plasma volume was defined based on the top 10 percentiles of study subjects.

The actual plasma volume (aPV), ideal plasma volume (iPV), and plasma volume status (PVS) were calculated by the following equations: aPV = (1-hematocrit)×[a+(b×weight (kg))], where hematocrit is a fraction (men: a = 1530 and b = 41; women, a = 864 and b = 47.9); iPV = c×weight (kg) where c = 39 in men and c = 40 in women; and PVS = [(aPV-iPV)/iPV]×100% [15–17]. The normal range of PVS has not yet been defined. High PVS was defined as PVS > 0% according to a previous report [18].

### Measurements

FBS, HbA1c, total cholesterol, HDL-C, LDL-C, and triglyceride levels were measured. All blood analyses were performed at a local laboratory. The methods for the analyses were not standardized between laboratories. However, the analyses were based on the Japan Society of Clinical Chemistry recommended methods for laboratory tests, which have been widely accepted by laboratories across Japan.

### Endpoint and follow-up

After obtaining permission from the Ministry of Health, Labour and Welfare, we accessed the database containing death certificates for all deaths that occurred between 2008 and 2015 in Japan. All subjects were prospectively followed for a median follow-up period of 3.9 years (interquartile range, 2.9–4.5 years; longest follow-up, 6 years). Prognostic outcomes were censored from the date of second health check-up. Subjects who survived were followed until they were 75-years old. The endpoints were cardiovascular death, non-cardiovascular death, and all-cause death. The cause of death was determined by reviewing the death certificates and classified based on the death code (International Classification of Diseases, 10^th^ Revision). The cardiovascular and non-cardiovascular deaths were defined as the death codes including I00 to I99 and the remaining death codes, respectively. The main causes of non-cardiovascular death were cancer, pulmonary disease, and gastrointestinal disorders.

### Statistical analysis

The normality of continuous variables was checked using a Kolmogorov–Smirnov–Lillefors test. The correlations of 1-year change in plasma volume with baseline actual PV and PVS were assessed by simple linear analysis. Continuous and categorical variables were compared using t-tests and chi-square tests, respectively. Survival curves were constructed using the Kaplan–Meier method and compared using log-rank tests. Logistic analysis was performed to determine independent risk factors for abnormally large 1-year expansion in plasma volume, and significant predictors selected in univariate analysis were entered into the multivariate analysis. Cox proportional hazard analysis was performed to determine independent predictors for all-cause death and cardiovascular risk factors, and significant predictors selected in univariate analysis and cardiovascular risk factors were entered into the multivariate analysis. Receiver operating characteristic (ROC) curves for all-cause deaths were constructed and used as a measure of the predictive accuracy of 1-year change in plasma volume for all-cause deaths. We calculated the net reclassification index (NRI) and integrated discrimination index (IDI) to measure the quality of improvement for the correct reclassification by the addition of 1-year change in plasma volume to the multivariate model. P-values < 0.05 were considered statistically significant. All statistical analyses were performed using standard statistical program packages (JMP version 12; SAS Institute Inc., Cary, NC, USA) and R version 3.0.2, with additional packages including Rcmdr, Epi, pROC, and PredictABEL.

## Results

### Baseline characteristics of study subjects

The baseline characteristics of the subjects are shown in Table 1.

**Table 1:** Baseline clinical characteristics in all subjects.

Variables	All subjects n = 134,291
Age, years	65 ± 7
Male, n (%)	51,309 (38%)
BMI, kg/m^2^	22.8 ± 3.2
Hypertension, n (%)	61,862 (46%)
Systolic BP, mmHg	129 ± 17
Diastolic BP, mmHg	76 ± 11
Dyslipidemia, n (%)	74,937 (56%)
Diabetes mellitus, n (%)	14,109 (10.5%)
Smoking, n (%)	15,857 (12%)
Framingham risk score	6.8 ± 3.9
Previous cardiovascular disease, n (%)	8,580 (6.4%)
Previous cerebrovascular disease, n (%)	5,035 (3.7%)
Previous anemia, n (%)	18,647 (13.9%)
Region area	
Hokkaido and Tohoku	13,701 (10.2)
Kanto and Koshinetsu	64,999 (48.4)
Kinki, Shikoku and Chugoku	10,756 (8.0)
Kyushu and Okinawa	44,835 (33.4)
Baseline high PVS, n (%)	42,593 (32%)
*Biochemical data*	
Baseline actual PV, mL	2148 ± 279
Baseline PVS	-3.3 ± 7.7
Baseline Hb, g/dL	13.6 ± 1.4
Baseline Hematocrit, mg/dL	41.1 ± 3.8
HbA1c (%)	5.4 ± 0.6
FBS, mg/dL	97 ± 19
Total cholesterol, mg/dL	211 ± 36
Triglyceride, mg/dL	119 ± 76
HDL-C, mg/dL	62.6 ± 16.6
LDL-C, mg/dL	125 ± 31
Hb at 1 year, g/dL	13.6 ± 1.3
Hematocrit at 1 year, mg/dL	41.1 ± 3.8
1-year change in PV (%)	0.19 ± 7.2
*Medications*	
Anti-hypertensive drug, n (%)	42,970 (32%)
Anti-dyslipidemia drug, n (%)	25,316 (19%)
Anti-diabetic drug, n (%)	6,650 (5.0%)
Cardiovascular disease, n (%)	2,145 (1.6%)
Cerebrovascular disease, n (%)	1,334 (1.0%)

Data are expressed as mean ± SD or number (percentage). BMI, body mass index; BP, blood pressure; FBS, fasting blood sugar; Hb, hemoglobin; HbA1c, glycosylated hemoglobin A1c; HDL-C, high-density lipoprotein cholesterol; LDL-C, low-density lipoprotein cholesterol; PV, plasma volume; PVS, plasma volume status.

**Fig 2:**
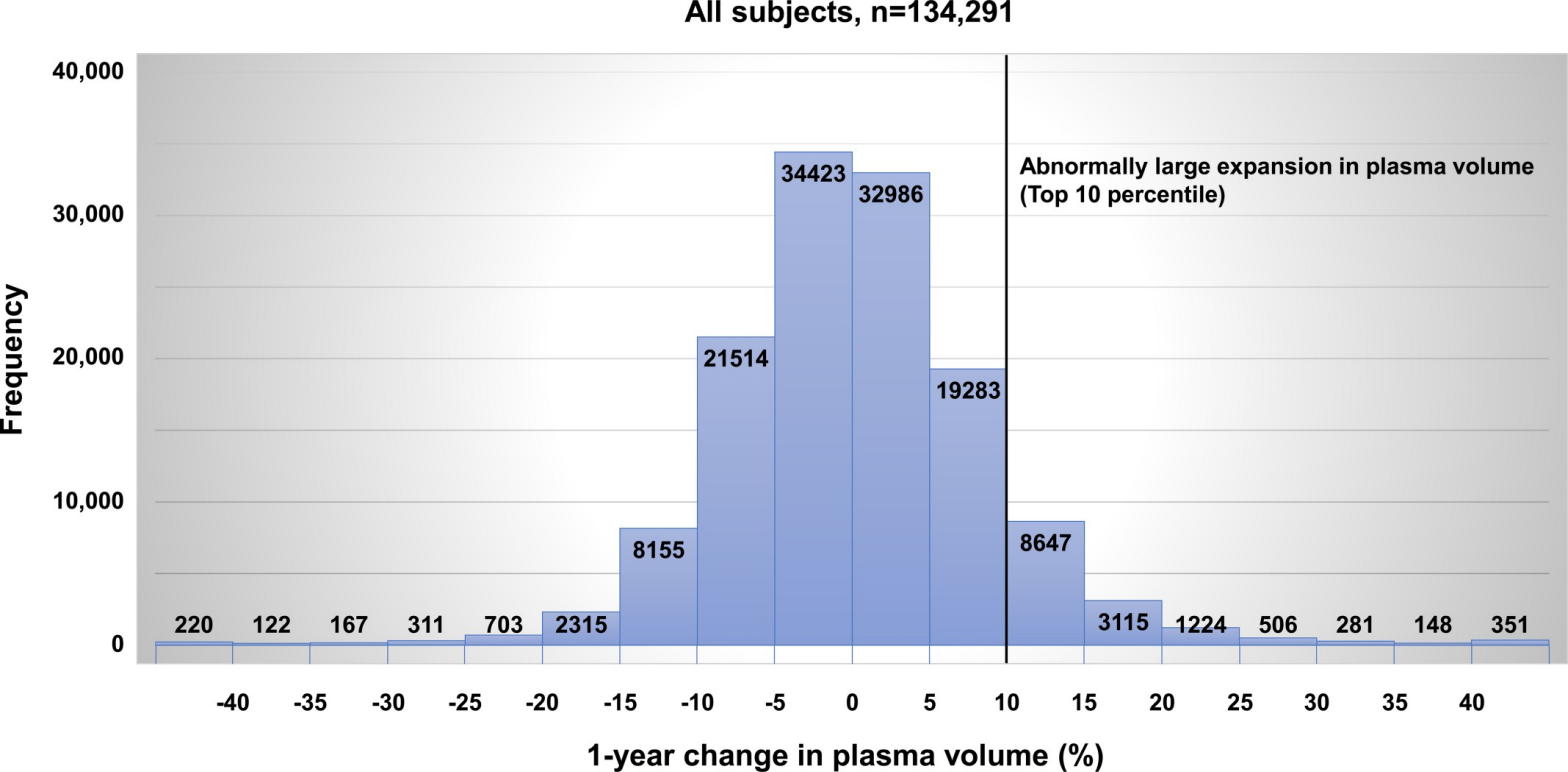
Association between frequency and 1-year change in plasma volume.

The mean 1-year change in plasma volume was 0.19%. The association between frequency and 1-year change in plasma volume is described in Fig 2. The 1-year change in plasma volume was normally distributed. Hypertension, dyslipidemia, and diabetes mellitus were identified in 61,862 (46%), 74,937 (56%), and 14,109 (10.5%) subjects, respectively, and 42,593 (32%) subjects had baseline high PVS. Previous cardiovascular disease, previous cerebrovascular disease, and previous anemia were identified in 8,580 (6.4%), 5,035 (3.7%), and 18,647 (13.9%) subjects, respectively. During the 1-year interval, new onsets of cardiovascular disease and cerebrovascular disease were identified in 2,145 (1.6%) and 1,334 (1.0%) subjects, respectively.

All subjects were divided into quintiles according to the 1-year change in plasma volume: first quintile (≤ -6.20%, n = 26,860), second quintile (-6.19% to -1.99%, n = 26,861), third quintile (-1.98% to 1.69%, n = 26,925), fourth quintile (1.70% to 6.35%, n = 26,798), and fifth quintile (> 6.35%, n = 26,847). The fifth quintile group was more likely to be male; to have hypertension, dyslipidemia, diabetes mellitus, and previous cardiovascular disease; to be current smokers; or be taking anti-hypertensive or anti-diabetic drugs compared to the remaining four groups. The prevalence of baseline high PVS in the fifth quintile was lowest among the five groups. In addition, the fifth quintile group showed higher levels of systolic and diastolic blood pressure, Framingham risk score, baseline hemoglobin, baseline hematocrit, FBS, HbA1c, total cholesterol, and triglyceride, and lower levels of baseline aPV, baseline PVS, hemoglobin at 1 year, and hematocrit at 1 year compared to the other four groups. HDL-C and LDL-C levels were higher in the fifth quintile group than in the first to third quintile groups. The fifth quintile group had higher prevalence of previous anemia than second to fourth quintiles. The fifth quintile group was older and had a higher body mass index level than the first quintile group (Table 2).

**Table 2:** Comparison of clinical characteristics among quintiles based on 1-year change in plasma volume.

Variables	1^st^ quintile	2^nd^ quintile	3^rd^ quintile	4^th^ quintile	5^th^ quintile
	≤ -6.20	-6.19~-1.99	-1.98~1.69	1.70~6.35	> 6.35
	n = 26,860	n = 26,861	n = 26,925	n = 26,798	n = 26,847
Age, years	64 ± 8	65 ± 7*	65 ± 7*	65 ± 7*	65 ± 7*
Male, n (%)	10,750 (40%)	9,519 (35%)	9,550 (35%)	9,913 (37%)	11,577 (43%)||
BMI, kg/m^2^	22.7 ± 3.2	22.9 ± 3.2*	22.9 ± 3.2*	22.9 ± 3.2*	22.8 ± 3.2*
Hypertension, n (%)	11,769 (44%)	12,064 (45%)	12,394 (46%)	12,435 (46%)	13,200 (49%)||
Systolic BP, mmHg	127 ± 16	128 ± 16*	128 ± 16*	129 ± 17*†‡	130 ± 17*†‡§
Diastolic BP, mmHg	75 ± 11	76 ± 10*	76 ± 11*	76 ± 11*†‡	77 ± 11*†‡§
Dyslipidemia, n (%)	13,641 (51%)	14,774 (55%)	15,256 (57%)	15,445 (58%)	15,821 (59%)||
Diabetes mellitus, n (%)	2,728 (10.2%)	2,528 (9.4%)	2,639 (9.8%)	2,784 (10.4%)	3,430 (12.8%)||
Smoking, n (%)	3,413 (12.7%)	2,859 (10.6%)	2,811 (10.4%)	2,973 (11.1%)	3,801 (14.2%)||
Framingham risk score	6.3 ± 4.0	6.7 ± 3.8*	6.9 ± 3.7*†	7.0 ± 3.8*†‡	7.1 ± 3.9*†‡§
Previous cardiovascular disease, n (%)	1,833 (6.9%)	1,604 (6.1%)	1,634 (6.2%)	1,624 (6.1%)	1,885 (7.1%)||
Previous cerebrovascular disease, n (%)	1,030 (3.9%)	990 (3.7%)	954 (3.6%)	1,005 (3.8%)	1,056 (4.0%)
Previous anemia, n (%)	4,271 (15.9%)	3,546 (13.2%)	3,505 (13.1)	3,518 (13.1%)	3,807 (14.2%)||
Region area					
Hokkaido and Tohoku	2,550 (9.5%)	2,639 (9.8%)	2,676 (9.9%)	2,804 (10.5%)	3,032 (11.3%)||
Kanto and Koshinetsu	12,856 (47.9%)	13,213 (49.2%)	13,281 (49.3%)	13,048 (48.7%)	12,601 (46.9%)
Kinki, Shikoku and Chugoku	1,945 (7.2%)	2,159 (8.0%)	2,084 (7.7%)	2,293 (8.6%)	2,275 (8.5%)
Kyushu and Okinawa	9,509 (35.4%)	8,850 (33.0%)	8,884 (33.0%)	8,653 (32.3%)	8,939 (33.3%)
Baseline high PVS, n (%)	11,894 (44%)	9,034 (34%)	8,218 (31%)	7,154 (27%)	6,293 (23%)||
*Biochemical data*					
Baseline actual PV, mL	2203 ± 292	2153 ± 273*	2140 ± 271*†	2128 ± 272*†‡	2116 ± 278*†‡§
Baseline PVS	-0.8 ± 8.2	-2.8 ± 7.2*	-3.4 ± 7.3*†	-4.3 ± 7.3*†‡	-5.3 ± 8.1*†‡§
Baseline Hb, g/dL	13.1 ± 1.5	13.5 ± 1.2*	13.6 ± 1.2*†	13.7 ± 1.3*†‡	14.0 ± 1.4*†‡§
Baseline Hematocrit, mg/dL	39.7 ± 4.1	40.7 ± 3.5*	41.1 ± 3.5*†	41.6 ± 3.6*†‡	42.5 ± 4.0*†‡§
HbA1c (%)	5.35 ± 0.61	5.36 ± 0.59	5.36 ± 0.58	5.38 ± 0.63*†‡	5.43 ± 0.77*†‡§
FBS, mg/dL	96 ± 18	95 ± 18	96 ± 17	97 ± 19*†‡	99 ± 23*†‡§
Total cholesterol, mg/dL	204 ± 34	210 ± 34*	212 ± 33*†	214 ± 36*†‡	216 ± 40*†‡§
Triglyceride, mg/dL	115 ± 73	117 ± 72	118 ± 72*	120 ± 75*†‡	126 ± 86*†‡§
HDL-C, mg/dL	61.3 ± 16.1	62.5 ± 16.1*	62.8 ± 16.2*	63.2 ± 16.7*†	63.2 ± 17.8*†‡
LDL-C, mg/dL	120 ± 29	124 ± 30*	125 ± 29*†	127 ± 31*†‡	127 ± 33*†‡
Hb at 1 year, g/dL	14.0 ± 1.4	13.8 ± 1.3*	13.6 ± 1.3*†	13.4 ± 1.3*†‡	13.1 ± 1.4*†‡§
Hematocrit at 1 year, mg/dL	42.6 ± 3.9	41.7 ± 3.5*	41.2 ± 3.5*†	40.7 ± 3.5*†‡	39.7 ± 4.1*†‡§
*Medications*					
Anti-hypertensive drug, n (%)	8,492 (31%)	8,465 (32%)	8,731 (32%)	8,475 (32%)	8,870 (33%)||
Anti-dyslipidemia drug, n (%)	4,771 (17.8%)	5,166 (19.2%)	5,315 (19.7%)	5,222 (19.5%)	4,842 (18.0%)||
Anti-diabetic drug, n (%)	1,347 (5.0%)	1,194 (4.5%)	1,244 (4.6%)	1,288 (4.8%)	1,577 (5.9%)||

Data are expressed as mean ± SD, number (percentage), or median.

BMI, body mass index; BP, blood pressure; FBS, fasting blood sugar; Hb, hemoglobin; HbA1c, glycosylated hemoglobin A1c; HDL-C, high-density lipoprotein cholesterol; LDL-C, low-density lipoprotein cholesterol; PV, plasma volume; PVS, plasma volume status.

*P<0.05 v.s. 1^st^ quintile,

†P<0.05 v.s. 2^nd^ quintile,

‡P<0.05 v.s. 3^rd^ quintile,

§P<0.05 v.s. 4^th^ quintile by analysis of variance with Turkey’s post hoc test, ||P<0.05 by chi-square test.

The fourth quintile group was more likely to have hypertension, dyslipidemia, and diabetes mellitus compared to the first to third quintile groups. In addition, the fourth quintile group showed higher levels of systolic and diastolic blood pressure, Framingham risk score, baseline hemoglobin, baseline hematocrit, FBS, HbA1c, total cholesterol, triglyceride, and LDL-C, and lower levels of baseline aPV, baseline PVS, hemoglobin at 1 year, and hematocrit at 1 year compared to the first to third quintile groups. HDL-C levels were higher in the fourth quintile group than in the first and second quintile groups. The fourth quintile group was older and had a higher body mass index level than the first quintile group (Table 2).

The third quintile group was more likely to have hypertension and dyslipidemia, or be taking anti-dyslipidemia drugs compared to the first and second quintile groups. In addition, the third quintile group showed higher Framingham risk score, baseline hemoglobin, baseline hematocrit, total cholesterol, and LDL-C levels, and lower levels of baseline aPV, baseline PVS, hemoglobin at 1 year, and hematocrit at 1 year compared to the first and second quintile groups. The third quintile was older and had higher body mass index, systolic and diastolic blood pressure, triglyceride, and HDL-C than the first quintile group (Table 2).

The second quintile group was older and more likely to have dyslipidemia or be taking anti-dyslipidemia drugs compared to the first quintile group. In addition, the second quintile group showed higher body mass index, systolic and diastolic blood pressure, Framingham risk score, higher levels of baseline hemoglobin, baseline hematocrit, total cholesterol, HDL-C, and LDL-C, and lower levels of baseline aPV, baseline PVS, hemoglobin at 1 year, and hematocrit at 1 year compared to the first quintile group (Table 2).

There was no significant difference in the incidence of previous cerebrovascular disease among groups.

**Fig 3:**
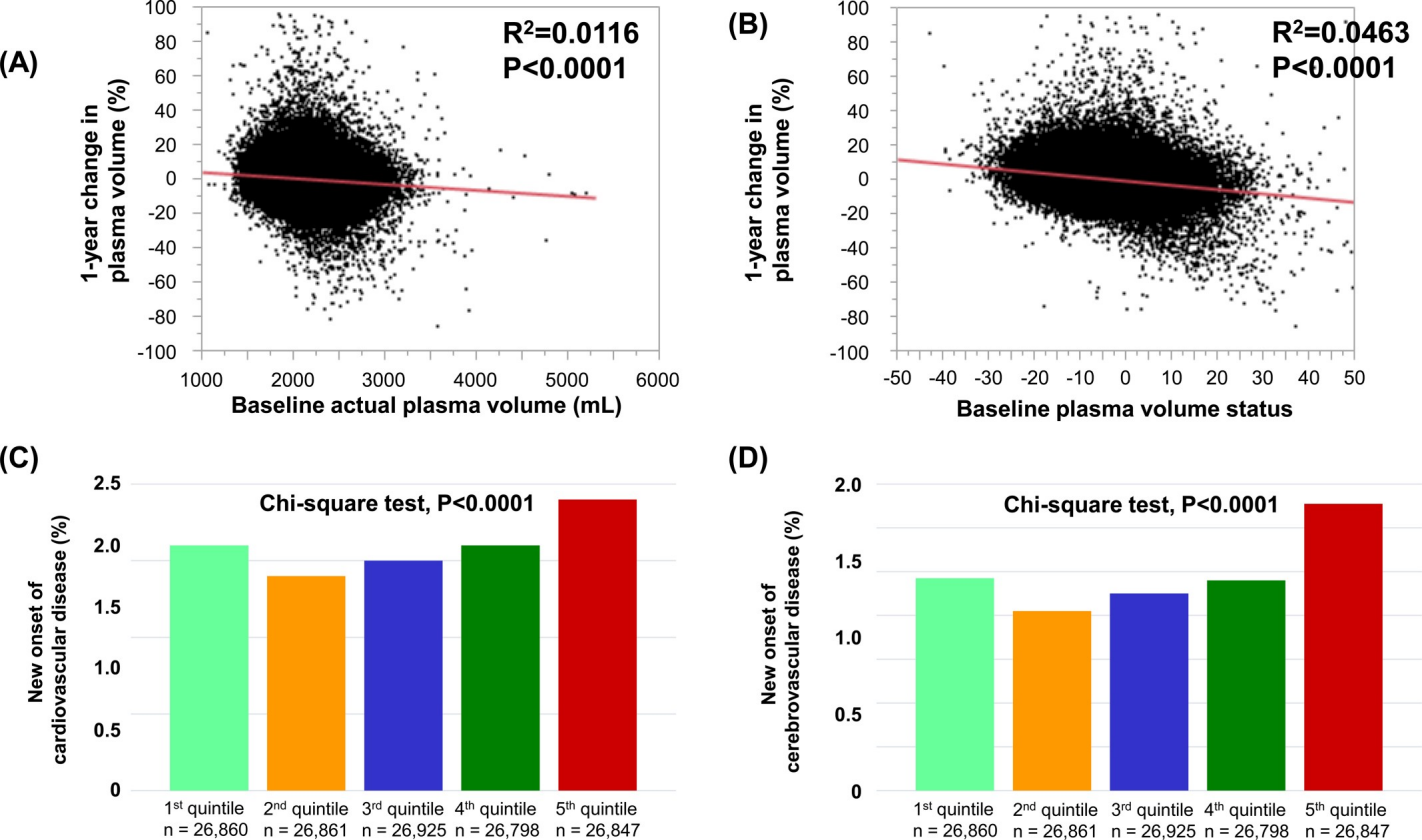
Association of 1-year change in plasma volume with baseline actual plasma volume (A) and plasma volume status (B). Comparisons of the rates of new onset cardiovascular disease (C) and cerebrovascular disease (D) among quintiles based on the 1-year change in plasma volume.

Simple linear analysis showed that a 1-year change in plasma volume was negatively correlated with baseline aPV and baseline PVS (Fig 3A and 3B). The incidences of cardiovascular disease and cerebrovascular disease were greatest in the highest fifth quintile group (Fig 3C and 3D).

### Risk for abnormally large 1-year expansion in plasma volume in the general population

Univariate and multivariate logistic analyses were performed to determine the risk factors for abnormally large 1-year expansion in plasma volume. In univariate logistic analysis, sex, hypertension, dyslipidemia, diabetes mellitus, smoking, Framingham risk score, previous cardiovascular disease, previous cerebrovascular disease, previous anemia, region area, and baseline PVS were related to abnormally large 1-year expansion in plasma volume (Table 3).

**Table 3:** Univariate and multivariate logistic analyses for abnormally 1-year large expansion in plasma volume.

Variables	Univariate			Multivariate		
	Odds ratio	95% CI	P value	Odds ratio	95% CI	P value
Age	1.000	0.997–1.003	0.7507			
Sex	1.477	1.426–1.530	<0.0001	1.337	1.322–1.428	<0.0001
BMI	0.704	0.448–1.107	0.1280			
Hypertension	1.213	1.172–1.257	<0.0001	1.104	1.062–1.147	<0.0001
Dyslipidemia	1.179	1.137–1.221	<0.0001	1.107	1.065–1.151	<0.001
Diabetes mellitus	1.479	1.404–1.555	<0.0001	1.311	1.241–1.386	<0.001
Smoking	1.418	1.350–1.489	<0.0001	1.224	1.161–1.288	<0.0001
Framingham risk score	1.023	1.020–1.030	<0.0001	1.000	0.994–1.005	0.8790
Previous cardiovascular disease	1.232	1.151–1.316	<0.0001	1.114	1.041–1.193	0.0020
Previous cerebrovascular disease	1.154	1.056–1.258	0.0016	1.035	0.946–1.132	0.4403
Previous anemia	1.126	1.071–1.189	<0.0001	1.415	1.344–1.490	<0.0001
Region area						
Hokkaido/Tohoku vs. Kanto/Koshinetsu	1.208	1.139–1.279	<0.0001	1.141	1.076–1.210	<0.0001
Kinki/Shikoku vs. Kanto/Koshinetsu	1.100	1.029–1.174	0.0048	1.116	1.043–1.195	0.0016
Kyushu/Okinawa vs. Kanto/Koshinetsu	1.051	1.011–1.094	0.0121	1.044	1.003–1.086	0.0370
Baseline high plasma volume status	0.609	0.585–0.635	<0.0001	0.636	0.609–0.664	<0.0001

BMI, body mass index; CI, confidence interval.

Multivariate logistic analysis demonstrated that sex, hypertension, dyslipidemia, diabetes mellitus, smoking, previous cardiovascular disease, previous anemia, region area, and baseline PVS were significantly associated with abnormally large 1-year expansion in plasma volume (Table 3).

### One-year change in plasma volume and mortality

The clinical characteristics at 1 year of the subjects are shown in Table 4.

**Table 4:** Clinical characteristics at 1 year in all subjects.

Variables	All subjects n = 134,291
Hypertension at 1 year, n (%)	63,113 (47%)
Dyslipidemia at 1 year, n (%)	87,188 (65%)
Diabetes mellitus at 1 year, n (%)	15,197 (11.3%)
Smoking at 1 year, n (%)	15,857 (12%)
Framingham risk score at 1 year	6.9 ± 3.2
Previous cardiovascular disease at 1 year, n (%)	10,725 (8.0%)
Previous anemia at 1 year, n (%)	19,393 (14.4%)
High plasma volume status at 1 year, n (%)	42,179 (31%)

Data are expressed as mean ± SD, number (percentage), or median.

**Fig 4:**
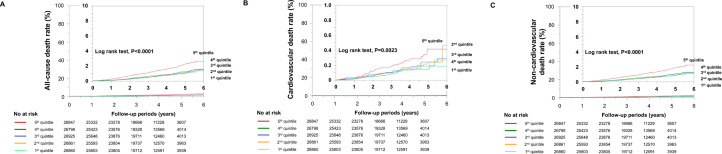
Kaplan–Meier analysis of all-cause deaths (A), cardiovascular deaths (B), and non-cardiovascular deaths (C) among quintiles based on the 1-year change in plasma volume.

All subjects were prospectively followed during a median follow-up of 3.9 years. During the follow-up period, there were 220 cardiovascular deaths, 1,001 non-cardiovascular deaths including 718 cancer deaths, and 1,221 all-cause deaths. Kaplan–Meier analysis demonstrated that the highest fifth quintile had the greatest risk for all-cause, cardiovascular, and non-cardiovascular deaths among the five groups (Fig 4A–4C). We performed univariate and multivariate Cox proportional hazard regression analyses to determine the risk factors for predicting all-cause, cardiovascular, non-cardiovascular, and cancer deaths. In univariate analysis, a 1-year change in plasma volume was significantly associated with all-cause, cardiovascular, non-cardiovascular, and cancer deaths. In addition, age at 1 year, sex, region area, hypertension at 1 year, diabetes mellitus at 1 year, Framingham risk score at 1 year, and smoking at 1 year were related to all-cause, cardiovascular, non-cardiovascular, and cancer deaths (Table 5).

**Table 5:** Univariate and multivariate Cox proportional hazard analyses of predicting all-cause, cardiovascular, non-cardiovascular, and cancer deaths.

	*Univariate analysis*			*Multivariate analysis*		
Variables	HR	95% CI	P value	HR	95% CI	P value
***All-cause deaths***						
1-year change in PV (Per 1SD increase)	1.375	1.319–1.434	<0.0001	1.281	1.225–1.338	<0.0001
Age at 1 year (Per 1-year increase)	1.114	1.099–1.129	<0.0001	1.104	1.089–1.119	<0.0001
Men vs. women	2.874	2.558–3.232	<0.0001	2.313	2.037–2.629	<0.0001
Region area						
Hokkaido/Tohoku vs. Kanto/Koshinetsu	1.457	1.213–1.739	<0.0001	1.523	1.266–1.820	<0.0001
Kinki/Shikoku vs. Kanto/Koshinetsu	1.242	1.011–1.511	0.0388	1.322	1.076–1.610	0.0085
Kyushu/Okinawa vs. Kanto/Koshinetsu	0.539	0.458–0.632	<0.0001	0.609	0.517–0.715	<0.0001
Hypertension at 1 year	1.423	1.271–1.593	<0.0001	1.097	0.975–1.234	0.1229
Dyslipidemia at 1 year	0.906	0.807–1.019	0.0971	0.971	0.857–1.102	0.6492
Diabetes mellitus at 1 year	2.058	1.781–2.368	<0.0001	1.552	1.329–1.804	<0.0001
Framingham risk score at 1 year (Per 1 score increase)	1.051	1.035–1.068	<0.0001	0.984	0.964–1.004	0.1186
Previous cardiovascular disease at 1 year	1.486	1.210–1.804	0.0002	1.058	0.859–1.288	0.5896
Previous anemia at 1 year	0.882	0.744–1.039	0.1425	1.116	0.935–1.324	0.2194
Smoking at 1 year	1.976	1.723–2.259	<0.0001	1.659	1.430–1.919	<0.0001
High plasma volume status at 1 year	1.619	1.445–1.814	<0.0001	1.469	1.289–1.658	<0.0001
***Cardiovascular deaths***						
1-year change in PV (Per 1SD increase)	1.267	1.133–1.417	<0.0001	1.166	1.036–1.313	0.0110
Age at 1 year (Per 1-year increase)	1.190	1.147–1.235	<0.0001	1.155	1.112–1.201	<0.0001
Men vs. women	2.818	2.143–3.722	<0.0001	2.251	1.669–3.048	<0.0001
Region area						
Hokkaido/Tohoku vs. Kanto/Koshinetsu	1.629	1.083–2.383	0.0202	1.744	1.155–2.559	0.0091
Kinki/Shikoku vs. Kanto/Koshinetsu	0.926	0.528–1.515	0.7728	0.961	0.548–1.575	0.8819
Kyushu/Okinawa vs. Kanto/Koshinetsu	0.461	0.306–0.672	<0.0001	0.540	0.359–0.790	0.0012
Hypertension at 1 year	2.461	1.862–3.283	<0.0001	1.567	1.174–2.111	0.0021
Dyslipidemia at 1 year	1.306	0.981–1.759	0.0669	1.135	0.836–1.559	0.4198
Diabetes mellitus at 1 year	1.780	1.237–2.494	0.0012	0.964	0.653–1.389	0.8492
Framingham risk score at 1 year (Per 1 score increase)	1.128	1.088–1.170	<0.0001	1.063	1.012–1.118	0.0145
Previous cardiovascular disease at 1 year	3.248	2.257–4.552	<0.0001	2.238	1.545–3.161	<0.0001
Previous anemia at 1 year	0.653	0.409–0.991	0.0448	0.951	0.588–1.467	0.8287
Smoking at 1 year	1.939	1.394–2.648	<0.0001	1.604	1.122–2.259	0.0101
High plasma volume status at 1 year	1.245	0.944–1.641	0.1176	1.338	0.987–1.801	0.0603
***Non-cardiovascular deaths***						
1-year change in PV (Per 1SD increase)	1.400	1.336–1.460	<0.0001	1.301	1.241–1.364	<0.0001
Age at 1 year (Per 1-year increase)	1.101	1.086–1.117	<0.0001	1.096	1.081–1.112	<0.0001
Men vs. women	2.886	2.538–3.286	<0.0001	2.343	2.035–2.699	<0.0001
Region area						
Hokkaido/Tohoku vs. Kanto/Koshinetsu	1.415	1.150–1.723	0.0012	1.471	1.194–1.797	0.0004
Kinki/Shikoku vs. Kanto/Koshinetsu	1.316	1.053–1.628	0.0165	1.406	1.124–1.741	0.0032
Kyushu/Okinawa vs. Kanto/Koshinetsu	0.558	0.466–0.663	<0.0001	0.625	0.522–0.744	<0.0001
Hypertension at 1 year	1.269	1.121–1.437	0.0002	1.019	0.895–1.161	0.7706
Dyslipidemia at 1 year	0.840	0.740–0.955	0.0078	0.944	0.822–1.084	0.4165
Diabetes mellitus at 1 year	2.122	1.811–2.473	<0.0001	1.714	1.447–2.023	<0.0001
Framingham risk score at 1 year (Per 1 score increase)	1.035	1.018–1.052	<0.0001	0.968	0.947–0.990	0.0050
Previous cardiovascular disease at 1 year	1.140	0.883–1.446	0.2964	0.817	0.631–1.040	0.1027
Previous anemia at 1 year	0.936	0.778–1.116	0.4691	1.146	0.946–1.378	0.1616
Smoking at 1 year	1.984	1.705–2.299	<0.0001	1.669	1.417–1.959	<0.0001
High plasma volume status at 1 year	1.712	1.509–1.939	<0.0001	1.491	1.298–1.712	<0.0001
***Cancer deaths***						
1-year change in PV (Per 1SD increase)	1.456	1.388–1.528	<0.0001	1.373	1.304–1.446	<0.0001
Age at 1 year (Per 1-year increase)	1.120	1.100–1.139	<0.0001	1.115	1.095–1.134	<0.0001
Men vs. women	2.940	2.523–3.429	<0.0001	2.284	1.934–2.702	<0.0001
Region area						
Hokkaido/Tohoku vs. Kanto/Koshinetsu	1.362	1.063–1.725	0.0153	1.396	1.088–1.770	0.0094
Kinki/Shikoku vs. Kanto/Koshinetsu	1.267	0.970–1.630	0.0810	1.327	1.015–1.709	0.0388
Kyushu/Okinawa vs. Kanto/Koshinetsu	0.547	0.442–0.671	<0.0001	0.618	0.499–0.760	<0.0001
Hypertension at 1 year	1.287	1.111–1.491	0.0008	0.997	0.856–1.162	0.9707
Dyslipidemia at 1 year	0.881	0.757–1.025	0.1012	0.965	0.820–1.138	0.6768
Diabetes mellitus at 1 year	2.241	1.864–2.677	<0.0001	1.774	1.455–2.150	<0.0001
Framingham risk score at 1 year (Per 1 score increase)	1.047	1.026–1.068	<0.0001	0.969	0.943–0.995	0.0210
Previous cardiovascular disease at 1 year	1.048	0.764–1.399	0.7619	0.736	0.535–0.986	0.0401
Previous anemia at 1 year	0.871	0.695–1.077	0.2150	1.102	0.872–1.377	0.4059
Smoking at 1 year	2.046	1.713–2.429	<0.0001	1.741	1.437–2.102	<0.0001
High plasma volume status at 1 year	1.600	1.378–1.856	<0.0001	1.332	1.129–1.569	0.0007

CI, confidence interval; HR, hazard ratio; PV, plasma volume.

Multivariate Cox proportional hazard regression analysis demonstrated that a 1-year change in plasma volume was an independent predictor of future all-cause, cardiovascular, non-cardiovascular, and cancer deaths after adjustment for age at 1 year, sex, region area, hypertension at 1 year, dyslipidemia at 1 year, diabetes mellitus at 1 year, Framingham risk score at 1 year, previous cardiovascular disease at 1 year, previous anemia at 1 year, smoking at 1 year, and high PVS at 1 year (Table 5).

### Improvement of reclassification by addition of 1-year change in plasma volume to predict all-cause mortality

To examine whether model fit and discrimination improved with the addition of a 1-year change in plasma volume to predictors such as age at 1 year, sex, region area, hypertension at 1 year, dyslipidemia at 1 year, diabetes mellitus at 1 year, Framingham risk score at 1 year, previous cardiovascular disease at 1 year, previous anemia at 1 year, smoking at 1 year, and PVS at 1 year, we evaluated the improvement in the C index, NRI, and IDI. The ROC curve analysis demonstrated that the C index of the baseline model for all-cause mortality was significantly improved by the addition of a 1-year change in plasma volume. NRI and IDI were also significantly improved by the addition of a 1-year change in plasma volume (Table 6).

**Table 6:** Statistics for model fit and improvement with the addition of 1-year change in plasma volume on the prediction of all-cause death.

	C index	NRI (95%CI, P value)	IDI (95%CI, P value)
***All-cause mortality***			
Baseline model	0.7494	Reference	Reference
Baseline model+1-year change in plasma volume	0.7596 (P = 0.0003)	0.1306 (0.0743–0.1869, P<0.0001)	0.0020 (0.0012–0.0027, P<0.0001)

Baseline model includes age at 1 year, sex, region area, hypertension at 1 year, dyslipidemia at 1 year, diabetes mellitus at 1 year, Framingham risk score at 1 year, previous cardiovascular disease at 1 year, previous anemia at 1 year, smoking at 1 year, and PVS at 1 year. IDI, integrated discrimination index; NRI, net reclassification index; 95%CI, 95% confidence interval.

## Discussion

The main findings in the present study were as follows: (1) A 1-year change in plasma volume was normally distributed and negatively correlated with baseline aPV and PVS; (2) risks for abnormally large 1-year expansion in plasma volume were cardiovascular risk factors and a lower baseline PVS, but not age and body mass index; (3) Kaplan–Meier analysis demonstrated that the highest quintile had the greatest risk for all-cause, cardiovascular, and non-cardiovascular deaths among the five groups; (4) multivariate analysis demonstrated that a 1-year change in plasma volume was an independent predictor of all-cause, cardiovascular, and non-cardiovascular deaths; and (5) the addition of 1-year change in plasma volume to other risk factors improved the prediction of all-cause deaths in the general population.

The prognostic value of 1-year change in plasma volume has not been examined in the general population until now. The clinical application of 1-year change in plasma volume is mainly discussed in the field of heart failure. The present study extended the previous studies regarding 1-year changes in plasma volume, and can provide new insight into the possibility that a 1-year change in plasma volume is a feasible marker for early identification of high-risk subjects in the general population.

### Plasma volume expansion and cardiovascular risk factors

The association between 1-year change in plasma volume and cardiovascular risk factors has not yet been fully examined. Hudson et al. showed that there was no significant difference in the prevalence of cardiovascular risk factors among three groups divided by changes in plasma volume tertiles in patients with heart failure [9]. In contrast, we showed that baseline PVS, previous cardiovascular disease, and cardiovascular risk factors, such as hypertension, dyslipidemia, diabetes mellitus, and smoking were independently related to abnormally large 1-year expansion in plasma volume in the general population. Moreover, there is a close relationship between plasma volume and the renin angiotensin aldosterone system. It is well known that renin secretion from juxta glomerular cells is augmented by reduction in plasma volume; this leads to renin angiotensin aldosterone system activation in an attempt to reduce renal excretion of sodium, thus tending to restore plasma volume by increasing blood osmolality [19]. Therefore, the inverse relationship between PVS and abnormally large 1-year change in plasma volume may be explained by the renin angiotensin aldosterone system in the general population.

The renin angiotensin aldosterone system is a major pathophysiology in the development of hypertension and cardiovascular disease [20]. Hyperglycemia has been reported to increase renin release in the kidney, and serum angiotensin converting enzyme activity is increased in patients with diabetes mellitus compared to healthy subjects [21], suggesting activation of the renin angiotensin aldosterone system in these subjects. Moreover, hypercholesterolemia is considered a risk for hypertension through renin angiotensin system activation [22]. It has been reported that cigarette smoking or nicotine inhalation in human volunteers led to an acute increase in both systolic and diastolic blood pressure, accompanied by increased plasma angiotensin converting enzyme activity [23]. In light of these findings, an abnormally large 1-year expansion in plasma volume may result from renin angiotensin aldosterone system activation due to a reduction in plasma volume, hypertension, dyslipidemia, diabetes mellitus, and smoking.

### One-year change in plasma volume and mortality

Accumulating evidence has demonstrated that plasma volume is a promising prognostic marker in patients with heart failure. Indeed, the prognostic value of a change in plasma volume has been previously reported in patients with heart failure [8, 9]. A large expansion in plasma volume greater than 29 was associated with all-cause and cardiovascular mortality in patients with heart failure with preserved ejection fraction in Asia [14]. The precise pathophysiological mechanism by which a large 1-year expansion in plasma volume may contribute to an increase in the risk of all-cause, cardiovascular, and non-cardiovascular mortality (mainly, cancer death) is unclear. A potential explanation is that volume overload caused by plasma volume expansion may exacerbate cardiac function. Alternatively, previous reports have suggested that neurohumoral activation, such as that of the renin angiotensin aldosterone system and sympathetic nervous activation, exacerbates cardiac function, leading to poor prognosis in heart failure patients [24]. Since this is an observational study, it is beyond the scope of the study to determine the causal relationship between 1-year change in plasma volume and mortality in the general population.

### Clinical perspectives

The clinical perspective of the present study was that a 1-year change in plasma volume is associated with all-cause death, indicating that subjects with large 1-year expansion in plasma volume need further examination for fatal diseases such as cancer, cardiovascular disease, and pulmonary disease. We have reported that PVS is a risk factor for mortality in the general population [25]. Interestingly, 1-year change in plasma volume predicted mortality in the general population independently of high PVS at 1 year, indicating the usefulness of serial plasma volume assessment. Importantly, the addition of a 1-year change in plasma volume to the established risk factors, including PVS at 1 year improved c-statistics, NRI, and IDI, indicate that it could be useful for the prevention, early identification, and management of potentially fatal diseases. There are several candidates for optimizing plasma volume therapy, such as renin angiotensin aldosterone inhibitors, ferric carboxymaltose, and sodium-glucose cotransporter 2 inhibitors in patients with heart failure [16, 26–28]. Future studies are required to examine whether changes in plasma volume-guided medication prevent premature deaths in the general population.

### Strengths and limitations

The strengths of the present study include its large sample size, prospective follow-up design, and nationwide data source. Therefore, our results are well generalized and highly reliable. However, there are also some limitations. First, we did not examine the incidence of non-fatal and infectious diseases. Thus, we could not determine the impact of a 1-year change in plasma volume on the development of non-fatal and infectious diseases. Second, we did not compare the calculated plasma volume, and instead measured plasma volume directly in the general population.

## Conclusion

This is the first report to reveal the impact of a 1-year change in plasma volume on all-cause, cardiovascular, and non-cardiovascular mortality. A 1-year change in plasma volume could be a feasible marker for all-cause, cardiovascular and non-cardiovascular (mainly cancer) mortality in the general population.
